# Implementation outcomes and strategies for delivering evidence-based hypertension interventions in lower-middle-income countries: Evidence from a multi-country consortium for hypertension control

**DOI:** 10.1371/journal.pone.0286204

**Published:** 2023-05-25

**Authors:** Joyce Gyamfi, Juliet Iwelunmor, Shivani Patel, Vilma Irazola, Angela Aifah, Ashlin Rakhra, Mark Butler, Rajesh Vedanthan, Giang Nguyen Hoang, Monicah Nyambura, Hoa Nguyen, Cuc Nguyen, Kwaku Poku Asante, Solomon Nyame, Kwame Adjei, John Amoah, Kingsley Apusiga, Kezia Gladys Amaning Adjei, Manuel Ramierz-Zea, Diego Hernandez, Meredith Fort, Hanspria Sharma, Prashant Jarhyan, Emmanuel Peprah, Gbenga Ogedegbe

**Affiliations:** 1 School of Global Public Health, New York University, New York, New York, United States of America; 2 Saint Louis University, Saint Louis, Missouri, United States of America; 3 Department of Global Health, Emory University, Atlanta, Georgia, United States of America; 4 Institute for Clinical Effectiveness and Health Policy (IECS), Buenos Aires, Argentina; 5 Section for Global Health, Institute for Excellence in Health Equity, NYU Grossman School of Medicine, New York, New York, United States of America; 6 Center for Personalized Health, Feinstein Institutes for Medical Research, Northwell Health, New York, New York, United States of America; 7 Health Strategy and Policy Institute, Vietnam Ministry of Health, Hanoi, Vietnam; 8 USAID AMPATH Kenya, Eldoret, Kenya; 9 Department of Population and Quantitative Health Sciences, University of Massachusetts Medical School, Worchester, Massachusetts, United States of America; 10 Kintampo Health Research Centre, Kintampo, Ghana; 11 Department of Physiology, Kwame Nkrumah University of Science and Technology (KNUST), Kumasi, Ghana; 12 Institute of Nutrition of Central America and Panama (INCAP), Guatemala City, Guatemala, United States of America; 13 Department of Health Systems, Management & Policy, Colorado School of Public Health, Aurora, Colorado, United States of America; 14 All India Institute of Medical Sciences, New Delhi, India; 15 Centre for Chronic Disease Control, New Delhi, India; Ensign Global College, GHANA

## Abstract

Guidance on contextually tailored implementation strategies for the prevention, treatment, and control of hypertension is limited in lower-middle income countries (Lower-MIC). To address this limitation, we compiled implementation strategies and accompanying outcomes of evidence-based hypertension interventions currently being implemented in five Lower-MIC. The Global Research on Implementation and Translation Science (GRIT) Coordinating Center (CC) (GRIT-CC) engaged its global network sites at Ghana, Guatemala, India, Kenya, and Vietnam. Purposively sampled implementation science experts completed an electronic survey assessing implementation outcomes, in addition to implementation strategies used in their ongoing hypertension interventions from among 73 strategies within the Expert Recommendations for Implementing Change (ERIC). Experts rated the strategies based on highest priority to their interventions. We analyzed the data by sorting implementation strategies utilized by sites into one of the nine domains in ERIC and summarized the data using frequencies, proportions, and means. Seventeen implementation experts (52.9% men) participated in the exercise. Of Proctor’s implementation outcomes identified across sites, all outcomes except for appropriateness were broadly assessed by three or more countries. Overall, 59 out of 73 (81%) strategies were being utilized in the five countries. The highest priority implementation strategies utilized across all five countries focused on evaluative and iterative strategies (e.g., identification of context specific barriers and facilitators) to delivery of patient- and community-level interventions, while the lowest priority was use of financial and infrastructure change strategies. More capacity building strategies (developing stakeholder interrelationships, training and educating stakeholders, and supporting clinicians) were incorporated into interventions implemented in India and Vietnam than Ghana, Kenya, and Guatemala. Although robust implementation strategies are being used in Lower -MICs, there is minimum use of financial and infrastructure change strategies. Our study contributes to the growing literature that demonstrates the use of Expert Recommendations for Implementing Change (ERIC) implementation strategies to deliver evidence-based hypertension interventions in Lower-MICs and will inform future cross-country data harmonization activities in resource-constrained settings.

## Introduction

Gaps still exist between implementing evidence-based hypertension interventions and improving effectiveness outcomes for patients in lower-middle income countries (Lower-MIC) [1]. Increasingly, scholars argue that the use of implementation strategies that are essential to overcome barriers and enhance the adoption, implementation and sustainment of evidence-based hypertension interventions in real-world settings is suboptimal [2–4]. The aforementioned issue is particularly salient in Lower-MIC context [3] where hypertension, remains a major public health issue, contributing to more than 80% of premature deaths [5]. The rationale for focusing on implementation strategies includes clarifying which methods or techniques were used to enhance the adoption, implementation, and sustainability of evidence-based hypertension interventions in Lower-MIC [6], and their potential scale-up to address the huge burden of hypertension in low-and-middle income countries [7].

For effective implementation of evidence-based interventions for hypertension control, purposively selected, context-sensitive implementation strategies are necessary. Further, reporting of implementation strategies should be consistently standardized across programs to facilitate replication and the ease of evaluating what works, for whom, and in what context [8–11]. The Expert Recommendations for Implementing Change (ERIC) has identified 73 discrete implementation strategies grouped into nine domains to better define and classify implementation strategies [12, 13]. ERIC has been applied across programs in the United States [14], but evidence of application of these strategies in Lower-MIC is limited. Thus, to address this gap, the objective of this study was to leverage the National Heart Lung and Blood Institute (NHLBI) funded consortium’s five Hypertension Outcomes for T4 Research (Hy-TREC) programs research teams to compile implementation strategies, and outcomes, across community and clinic-based intervention programs being conducted in Ghana [15], Guatemala [16], India [17], Kenya [18], and Vietnam [19]. This process was guided by Expert Recommendations for Implementing Change (ERIC) [12, 13] checklist, and specified the various components of the implementation strategies suggested by Proctor et al. [20]. We further delineated the implementation outcomes using Reach, Efficacy/Effectiveness, Adoption, Implementation, Maintenance (RE-AIM) framework [21] and assessed the Proctor’s implementation outcomes [22] being measured by each program. By detailing common implementation strategies among the Hy-TREC programs, we will advance the field of late-stage implementation science research and provide guidance for researchers, practitioners, and policymakers when scaling up evidence-based interventions for hypertension control in the community and routine clinical practice across resource-constrained settings worldwide.

## Methods

### Conceptual framework

The study design, methods, and analysis integrate the Expert Recommendations for Implementing Change (ERIC) checklist of suggested implementation strategies, Proctor’s implementation outcomes [23] and the RE-AIM framework [21, 24–26]. Our data collection tool was guided by the forementioned frameworks (see S1 File). ERIC was created to address gaps in the transfer of implementation science knowledge from research to practice by improving comprehensiveness, conceptual clarity, and relevance of implementation strategies [12, 13]. ERIC is a compilation of discrete strategies systematically gathered from the input of a diverse range of stakeholders (Powell et al., 2015) [12]. This compilation is to serve as “building blocks” for constructing implementation strategies, assessing existing strategies, and improving specification and reporting in implementation research [12, 13]. Proctor’s implementation outcomes distinguish implementation outcomes from clinical treatment outcomes and service system outcomes [22, 23]. These outcomes–acceptability, adoption, appropriateness, feasibility, fidelity, implementation cost, penetration, and sustainability- serve as proximal indicators for the implementation process, key intermediate outcomes in treatment effectiveness and quality of care, and indicators for implementation success [22, 23]. The RE-AIM framework assess five dimensions–reach, efficacy, adoption, implementation, and maintenance—to evaluate the impact of an intervention [21]. The framework is applicable at multiple levels (individual, clinic, organization, community), and can be used to fulfill diverse evaluative purposes (e.g., overall public health impact, comparing interventions across dimensions, etc.) [21]. We compiled data from each of the five Hy-TREC consortium member sites, with data collection occurring between October and November 2020.

### Study context

Hy-TREC is comprised of five single-country studies (4 trials and one quasi-experimental study) designed to evaluate implementation strategies and implementation outcomes for evidence-based interventions targeted at the prevention, treatment and control of hypertension in their respective settings. The intervention programs are being implemented over five years in Kenya, Ghana, Guatemala, India and Vietnam. Details of the Hy-TREC programs from the various countries are published elsewhere [15–19]. The Global Research on Implementation and Translation Science (GRIT) Coordinating Center (CC) [27–29] (GRIT-CC) was established to coordinate activities and synthesize knowledge across the separately funded sites. This study was conducted as a preparatory activity towards data harmonization and facilitate cross-country analyses.

### Overview of the Hy-TREC programs

Table 1 provides a brief overview of each Hy-TREC site’s program and the implementation frameworks used by the various implementing countries (Ghana, Guatemala, India, Kenya, Vietnam). All programs except India employed a cluster-randomized design involving patients and community members. India’s integrated tracking, referral, electronic decision support, and care coordination (I-TREC) program used a quasi-experimental design within a single intervention block and a single comparison block. The interventions employed were multi-component and included multi-level stakeholders, with mixed methods evaluation designs. The interventions comprise:

- Practice facilitation for the adoption of Task-Strengthening Strategy for Hypertension (TASSH) to improve systolic blood pressure reduction among adults with uncontrolled hypertension (*Ghana*); [15]
- Team-based collaborative care, health provider education, health coaching sessions, home-based blood monitoring, and blood pressure audit and feedback(*Guatemala*); [16]
- An electronic case record form (eCRF) to consolidate and track patient information and referrals across the publicly-funded healthcare system; an electronic clinical decision support system (CDSS) to assist clinicians to provide tailored guideline-based care to patients; a revised workflow to ensure coordinated care within and across facilities; and enhanced non-communicable disease training for physicians and nurses (*India*); [17]
- Health information technology (HIT) and peer support intervention to improve referral completion, blood pressure reduction, and cardiovascular disease (CVD) risk reduction (*Kenya*); [18]
- Expanded community health worker services; home blood pressure (BP) self-monitoring; and a “storytelling intervention,” which consists of interactive, literacy-appropriate, and culturally sensitive multimedia storytelling modules for motivating behavior change (*Vietnam*) [19].

The primary outcomes for the country programs included implementation outcomes (e.g., adoption, fidelity) and effectiveness outcomes (i.e., change in systolic and diastolic blood pressure).

**Table 1:** GRIT consortium Hy-TREC programs.

Hy-TREC Site & Project Title	Brief Summary of project	Implementation Framework
**Ghana** [15] *Uptake of Task-Strengthening Strategy for Hypertension Control within Community Health Planning Services in Ghana*: *A Mixed Method Study*	The goal of this study is to evaluate, in a hybrid clinical effectiveness- implementation cluster design, the effect of practice facilitation (PF) on the uptake of an evidence-based Task Strengthening Strategy for Hypertension control (TASSH), among 700 adults who present to 70 Community- Based Health Planning Services (CHPs) zones with uncontrolled hypertension.	RE-AIM [21] CFIR [30]
**Guatemala** [16] *Implementing a Multicomponent Intervention to Improve Hypertension Control in Central America*. *A Cluster Randomized Trial in Guatemala*	A cluster randomized clinical trial to test the co-primary objectives: The effect of a multilevel and multicomponent intervention program on blood pressure (BP) control among Guatemalan hypertensive patients over an 18-month period. The acceptability, adoption, feasibility, fidelity, reach, and sustainability of implementing the intervention in patients, providers, and health districts.	RE-AIM [21] PRISM [31]
**India** [17] *Integrated Tracking*, *Referral*, *and Electronic Decision Support*, *and Care Coordination (I-TREC)*	The overall goal of this 5-year project is to adapt, implement, and evaluate an IT- enabled platform for integrated tracking, referral, electronic decision support, and care coordination (I-TREC) to treat hypertension and diabetes in rural communities that rely on public health care system using mixed methods approach. (Quasi-experimental design)	RE-AIM [21]
**Kenya** [18] *Strengthening Referral Networks for Management of Hypertension Across the Health System (STRENGTHS) in western Kenya*: *a study protocol of a cluster randomized trial*	A cluster randomized control trial evaluating the effectiveness and cost- effectiveness of a combined health information technology (HIT) and peer support intervention on referral completion, BP improvement, and CVD risk reduction in Kenya.	Saunders [32] PRECEDE-PROCEED [33, 34]
**Vietnam** [19] *Conquering Hypertension in Vietnam*: *Solutions at Grassroots level*	A cluster randomized controlled trial to evaluate the implementation and effectiveness of two multi-faceted community and clinic-based strategies for the control of hypertension among adults residing in the rural Red River Delta region of Vietnam with uncontrolled hypertension.	RE-AIM [21]

RE-AIM [21]: Reach, Effectiveness, Adoption, Implementation, Maintenance

CFIR [30]: Consolidated Framework for Implementation Research

PRISM [31]: Practical, Robust Implementation and Sustainability Model

### Data collection

This study is a review of Hy-TREC site specific study protocols and synthesis of data. Expert study team members with experience in noncommunicable disease and implementation science provided verbal consent prior to data collection. Each Hy-TREC site received an electronic data form (See S1 File) template for the compilation of implementation outcomes, and implementation strategies to complete for their program in October 2020 (see measures section for additional details). Explicit instructions and definitions accompanied the form templates to ensure the standardization of reporting. Sites were encouraged to reach out to the GRIT-CC facilitator if they needed clarification for populating the forms with the requested information to be extracted from their proposed study protocol. The form was completed by at least 2–7 implementation experts from each site (17 total) and was received from all sites in November 2020. We used purposive sampling [35] to recruit implementation experts from each of the five country sites. The GRIT-CC consortium facilitator followed up with sites for clarification if there were any discrepancies between what sites reported in the study protocol and the completed form.

### Measures

**Implementation outcomes and other implementation measures** consists of constructs that are measures of intervention implementation identified by prior research and theory. Examples of implementation outcomes include adoption and feasibility (Tables 2 and 3). These outcomes are derived from multiple frameworks (e.g., RE-AIM, Proctor’s implementation outcomes, etc.) and are indicators of implementation processes and success. We identified the implementation outcomes that are being measured in each of the consortium projects. Also, to understand the implementation context, we collected data on other implementation measures including contextual factors, such as provider attitudes, professional behavior, and the service system and its impact on intervention implementation, implementation climate, leadership support, and organizational capacity.

**Table 2:** Implementation outcomes and other measures.

	Ghana	Guatemala	India	Kenya	Vietnam
**Proctor’s Implementation Outcomes**					
Acceptability		X	X	X	X
Adoption	X	X	X	X	X
Appropriateness				X	X
Costs		X	X	X	X
Feasibility		X	X	X	X
Fidelity	X	X	X	X	X
Sustainability	X	X	X		X
**RE-AIM Dimensions**					
Reach	X	X	X	X	X
Effectiveness	X	X	X	X	X
Adoption	X	X	X	X	X
Implementation	X	X	X	X	X
Maintenance	X	X	X		X
**Other Implementation measures**					
Context				X	
Implementation Climate	X		X	X	
Leadership Support	X		X	X	
Organizational Capacity	X			X	

**Table 3:** Implementation outcomes and other measures (information sources used).

Implementation Measures	General Definition	Information source used to capture measures across sites	Ghana	Guatemala	India	Kenya	Vietnam
**Proctor’s Implementation Outcomes**							
Acceptability	Extent to which implementation stakeholders perceive a treatment, service, practice, or innovation to be agreeable, palatable, or satisfactory	Surveys, patient and key informant interviews, semi-structured interviews, focus group discussions		X	X	X	X
Adoption	The absolute number, proportion, and representativeness of settings and intervention agents who are willing to initiate a program	Follow-up questionnaire on intervention adherence, intervention activities completion checklist, referral records, Focus groups, and Key informant interviews	X	X	X	X	X
Appropriateness	Perceived fit, relevance, or compatibility of the innovation or evidence-based practice for a given practice setting, provider, or consumer; and/or perceived fit of the innovation or evidence-based practice to address a particular issue or problem	Needs assessment, Focus group discussion with Clinicians /Administrators				X	X
Costs	Cost (incremental or implementation cost) is the cost impact of an implementation effort	Questionnaires and checklists to assess intervention/program and implementation cost, patient healthcare costs; and estimation of the incremental cost-effectiveness ratio		X	X	X	X
Feasibility	Extent to which a new innovation or practice can be successfully used or carried out within a given agency or setting	Focus group discussions, semi-structured interviews, surveys, intervention activities checklists, documentation of adaption done by implementers		X	X	X	X
Fidelity	Degree to which an intervention or implementation strategy was delivered as prescribed in the original protocol or as intended by program developers. May include multiple dimensions such as content, process, exposure, and dosage	Semi-structured interviews, field observation	X	X	X	X	X
Sustainability	The extent to which behavior is sustained 6 months or more after treatment and whether the program becomes part of the routine organizational practices and policies	Patient medical records, written evaluation, semi-structured interviews, focus group discussions, monitoring of post intervention activities	X	X	X		X
**RE-AIM Dimensions**							
Reach	The absolute number, proportion, and representativeness of individuals who are willing to participate in a given initiative, intervention or program	Screening and enrollment data	X	X	X	X	X
Effectiveness	The impact of the intervention on important outcomes	Differences in blood pressure measurement (SBP and DBP) at a pre-defined timepoint (Quantitative) and semi-structured interviews with adherent and nonadherent participants	X	X	X	X	X
Adoption	The absolute number, proportion, and representativeness of settings and intervention agents who are willing to initiate a program	Follow-up questionnaire on intervention adherence, intervention activities completion checklist, referral records	X	X	X	X	X
Implementation	At the setting level, implementation refers to the intervention agents’ fidelity to the various elements of an intervention’s protocol	Data measuring occurrence and quality of the various intervention components, Guideline-based treatment algorithms, field observation checklists, focus group discussions semi-structured interviews, costing tool	X	X	X	X	X
Maintenance	The extent to which behavior is sustained 6 months or more after treatment and whether the program becomes part of the routine organizational practices and policies		X	X	X		X
**Other Implementation Measures**							
Context	contextual factors, such as provider attitudes, professional behavior, and the service system	Process Evaluation Data: barriers and facilitators to implementing the intervention				X	
Implementation Climate	The extent to which employees perceive that the adoption, implementation, and use of an innovation such as Evidence-Based Practices (EBP) is expected, rewarded, and supported by the organization	Implementation Climate Scale (ICS)-18 items administered at baseline; Qualitative assessment of barriers, facilitators, contextual factors and readiness for change, Observational process mapping, Baseline referral network analysis	X		X	X	
Leadership Support	Leadership behaviors related to organizational culture and climate. Supportive leadership involves embedding mechanisms which promote strategic climates. Supportive leaders help their staff to implement Evidence-Based Practices (EBP)	Implementation Leadership Scale (ILS)-12 items, Qualitative assessment of barriers, facilitators, contextual factors and readiness for change, Observational process mapping, Baseline referral network analysis	X		X	X	
Organizational Capacity	The combination of managerial and organizational capabilities that allows an enterprise to adapt more quickly and effectively than its competition to changing situations	Organizational Capacity for Change (OCC)-32 items, Proficiency subscale of the Organizational Social Context Scale- 15 items, Qualitative assessment of barriers, facilitators, contextual factors and readiness for change, Observational process mapping, Baseline referral network analysis	X			X	

**General definitions** [21, 23, 30]

**Implementation Strategies** are specific actions being performed as part of the implementation process. We used the ERIC checklist of 73 different implementation strategies identified by implementation science experts [36]. We identified concrete strategies being implemented by study teams at their respective sites. Further, each country team provided detailed specifications for the implementation strategies that they are using. Proctor and colleagues [20], recommends providing specific details of how particular implementation strategies are operationalized. This includes defining important aspects of an implementation strategy (e.g., the actor who delivers the strategy, the action being targeted by the strategy and the dose of the strategy). By expanding upon the details of the implementation strategies which are most essential to the intervention, we can learn more about how the intervention is delivered and implementation fidelity.

### Data analysis

Data from all sites were merged into a Microsoft Excel spreadsheet and analysis was conducted using SPSS statistical software 26. We summarized the implementation outcomes and implementation strategies used across the studies. Details of the implementation strategies used by country sites were analyzed to evaluate common strategies used across country sites. The implementation strategies employed by sites were grouped under the following ERIC implementation strategies domains: 1) *Evaluative and iterative strategies*, *2) Provide interactive assistance*, *3) Adapt and tailor to context*, *4) Develop stakeholder interrelationships strategies*, *5) Train and educate stakeholder strategies*, *6) Strategies to support clinicians*, *7) Strategies to engage consumers*, *8) Financial strategies*, *and 9) Change of infrastructure strategies* [36].

**Fig 1:**
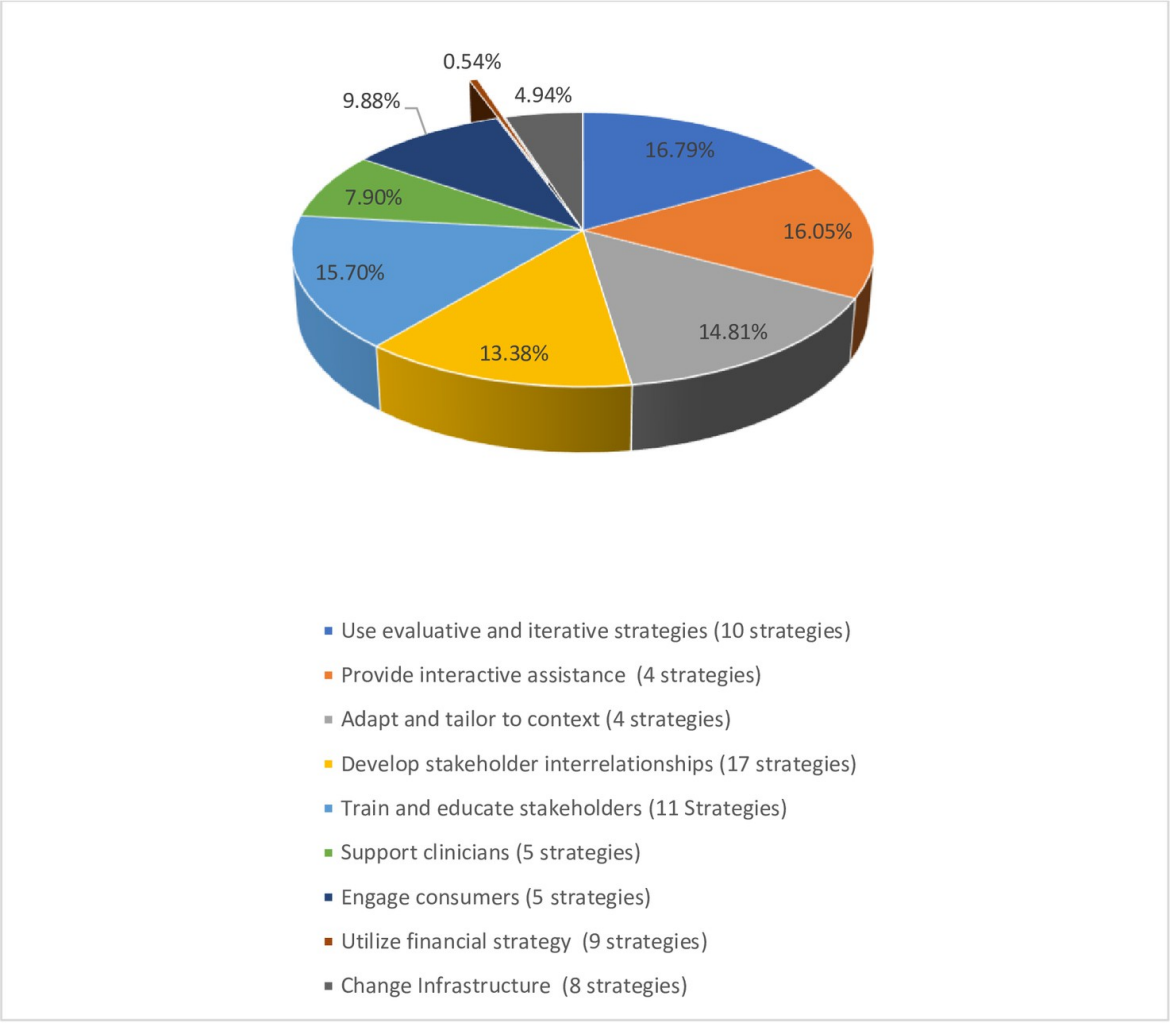
Implementation strategies used by Hy-TREC programs.

We summarized the implementation strategies using frequencies, proportions and means. We calculated the frequencies of programs using each of the ERIC strategies. From the count of country usage of strategies within each category, we obtained the average proportions of countries using specific implementation strategies within each category across the 5 programs (i.e., numerator = sum of countries using each strategy, denominator = 5 total countries). To provide a comparable summary score across programs, the percentage and number of programs utilizing each strategy as part of their intervention are reported. We also computed the overall average of strategies used per category by dividing the total sum within a category by the specified strategies for that category. The total overall averages were then summed across all categories. The overall averages per category was then divided by the category mean to obtain the proportion of strategies being used by sites from specific categories (Fig 1). Based on the reported data, summary tables and pie charts were constructed to display the distribution of the proportion of implementation strategies being used across the five programs. The data summaries were relayed back to expert respondents for feedback and confirmation. The GRIT-CC facilitator presented the summary findings to the group, explained the data synthesize process and answered questions from country teams regarding their specific country data. We then followed up with a detailed email to each team to revisit their study implementation details and make any updates to their study information as needed, and or confirm that we can move forward with the summary data.

## Results

### Characteristics of respondents

We synthesized implementation strategies, and implementation outcomes used across the five Hy-TREC programs (see Table 1). A total of 17 implementation experts participated, including 52.9% male, with over 50% of respondents between the ages of 35–44 years. Experts had on average 5.1 years of experience conducting implementation science research (SD: 2.6; range 2–10 years). Fifty-nine percent were doctoral level trained professionals and 35% were master’s level trained, with 6% being bachelor level trained. The roles held by the various experts on the project included investigators (42%), project managers (16%), and project coordinators (42%).

### Implementation outcomes, and other implementation measures

Implementation outcomes used by the Hy-TREC programs are described in Table 1 (studies are still ongoing). RE-AIM dimensions including reach, efficacy/effectiveness, adoption, implementation, are widely being assessed, with all countries reporting on these implementation measures (Tables 2 and 3). Four out of five countries will report on maintenance / sustainability. Table 3 provides the general implementation measure definitions and information sources used to capture RE-AIM dimensions and Proctor’s implementation outcomes across sites. Reach is measured by individual level of participation; Adoption is defined by sites as intention to use and or adherence to the intervention program; Acceptability (provider and patient attitudes) is measured as perceived acceptability by hypertensive patients, wider community, peer navigators, clinicians, and satisfaction with intervention and implementation process. Maintenance / sustainability is measured 12–24 months after conclusion of initial intervention.

Of the additional Proctor’s implementation outcomes identified across sites, all outcomes except for appropriateness are broadly being assessed by three or more countries, only two countries assessed appropriateness. All teams measured the adoption and fidelity of their programs to assess the uptake of the intervention and whether the study was implemented as intended. Appropriateness is being assessed by Kenya (for their integrated peer and technology referral support intervention) and Vietnam (for their community and clinic-based program involving community health workers and culturally tailored story telling interventions for blood pressure control, where participants were matched with local “stars” living in the same community with similar characteristics to help them through the behavior change process). Costs / cost-effectiveness is being assessed for the interventions implemented in Guatemala [16], India [17], Kenya [18], and Vietnam [19]. Specifically, Guatemala is evaluating the cost-effectiveness of the multilevel and multicomponent intervention program, compared to usual care [16]. Intervention costs include fixed costs such as education of health providers and salary of auxiliary nurses, and variable costs such as electronic BP monitors. Healthcare costs include ambulatory costs, such as drugs and laboratory tests, and hospital care (hospitalization). India is collecting data on patient expenditures related to outpatient and inpatient health care utilization in the last 3 months prior to survey completion to understand the cost incurred by patients to manage their hypertension [17].

The Kenyan team is evaluating the incremental cost-effectiveness of the combined HIT and peer support intervention, in terms of costs per unit decrease in SBP, per percent change in CVD risk score, and per disability-adjusted life year (DALY) saved [18]. In Vietnam, costs evaluations include the following: (1) program costs, which consist of costs to develop the intervention and implementation costs incurred at the district and community levels, and (2) patient costs for medications, diagnostic procedures, time lost, health center visits, and consultation fees [19].

The Hy-TREC studies used mixed-method approaches (qualitative and quantitative) for data collection. Reported data sources for evaluating the implementation outcomes include screening and enrollment data, questionnaires, semi-structured interviews, and focus groups. Implementation context, implementation climate, leadership support, and other organizational capacity was assessed via process evaluation and or the use of established implementation scales [37–39].

### Implementation strategies and components

Overall, 59 (81%) of the Expert Recommendations for Implementing Change (ERIC) suggested 73 strategies [36] were incorporated into the study protocols, with specific strategies being used by multiple country programs. In general, the use of evaluative and iterative strategies (e.g., needs assessment, identifying barriers and facilitators to implementation); providing interactive assistance, adaptation and tailoring the intervention to context, developing stakeholder interrelationships, and providing training and education to stakeholders, were the most prevalent strategies used among three or more country sites (average overall usage >50%) (Table 4). Country sites engaged both patients, implementers, and other key stakeholders (e.g., health policymakers) in the implementation process. Programs conducted in Asia (India and Vietnam) applied the most strategies with an average of 46 strategies used, while programs implemented in Africa (Ghana, Kenya) and Central America (Guatemala), used only about a third of the recommended implementation strategies, with an average of 21 and 24 strategies respectively used across sites. Strategies tailored at developing, training and educating stakeholders and supporting clinicians were less utilized in these settings. Fig 1 provides the overall proportion of strategies used by sites from the various domains. The sum of the country means from each of the nine ERIC categories was 20.2. On average, sites used only 0.1 (0.54%) of the nine strategies from the Use of financial incentives category; and a mean of 1 (4.94%) of strategies was used from the change of infrastructure category, which has 8 total strategies. We provide details of the implementation strategies, how the strategies were defined, how it was operationalized, and the affected implementation outcomes for each country’s program using the main implementation strategy as an example, in S1 Table. By expanding upon the details of the implementation strategies which are most essential to the intervention as done in S1 Table, we can learn more about how the intervention was delivered.

**Table 4:** Proportion of programs reporting implementation strategies and components.

Implementation Strategies	Frequency of programs using strategies	Proportion of programs using strategies
**Use evaluative and iterative strategies (10 strategies)**	**-**	
Assess for readiness and identify barriers and facilitators	**5**	100%
Audit and provide feedback	**4**	80%
Conduct local needs assessment	**4**	80%
Conduct cyclical small tests of change	**1**	20%
Develop and implement tools for quality monitoring	**4**	80%
Develop a formal implementation blueprint	**3**	60%
Develop and organize quality monitoring systems	**4**	80%
Purposely reexamine the implementation	**3**	60%
Obtain and use patients/consumers and family feedback	**4**	80%
Stage implementation scale up	**2**	40%
*Average of overall evaluative and iterative strategies	**3.4**	68%
**Provide interactive assistance (4 strategies)**		
Centralize technical assistance	**3**	60%
Facilitation	**4**	80%
Provide local technical assistance	**2**	40%
Provide clinical supervision	**4**	80%
*Average of overall provide interactive strategies	**3.3**	65%
**Adapt and tailor to context (4 strategies)**		
Promote adaptability	**2**	40%
Tailor strategies	**5**	100%
Use data experts	**4**	80%
Use data warehousing techniques	**1**	20%
*Average of overall adapt and tailor to context strategies	**3.0**	60%
**Develop stakeholder interrelationships (17 strategies)**		
Build a coalition	**3**	60%
Capture and share local knowledge	**4**	80%
Conduct local consensus discussions	**4**	80%
Develop an implementation glossary	**1**	20%
Develop academic partnerships	**3**	60%
Identify early adopters	**1**	20%
Identify and prepare champions	**2**	40%
Inform local opinion leaders	**3**	60%
Involve executive boards	**3**	60%
Model and simulate change	**2**	40%
Obtain formal commitments	**3**	60%
Organize clinician implementation team meetings	**2**	40%
Promote network weaving	**2**	40%
Recruit, designate, and train for leadership	**3**	60%
Use advisory boards and workgroups	**5**	100%
Use an implementation advisor	**5**	100%
Visit other sites	**0**	0%
*Average of overall develop stakeholder interrelationships strategies	**2.7**	54%
**Train and educate stakeholders (11 Strategies)**		
Conduct ongoing training	**4**	80%
Conduct educational meetings	**5**	100%
Conduct educational outreach visits	**4**	80%
Create a learning collaborative	**3**	60%
Develop educational materials	**4**	80%
Distribute educational materials	**5**	100%
Make training dynamic	**3**	60%
Provide ongoing consultation	**2**	40%
Shadow other experts	**1**	20%
Use train-the-trainer strategies	**2**	40%
Work with educational institutions	**2**	40%
*Average of overall train and educate stakeholders strategies	**3.2**	64%
**Support clinicians (5 strategies)**		
Create new clinical teams	**1**	20%
Develop resource sharing agreements	**3**	60%
Facilitate relay of clinical data to providers	**1**	20%
Remind clinicians	**2**	40%
Revise professional roles	**1**	20%
*Average of overall support clinicians’ strategies	**1.6**	32%
**Engage consumers (5 strategies)**		
Involve patients/consumers and family members	**4**	80%
Intervene with patients/consumers to enhance uptake and adherence	**4**	80%
Prepare patients/consumers to be active participants	**1**	20%
Increase demand	**0**	0%
Use mass media	**1**	20%
*Average of overall engage consumers strategies	**2.0**	40%
**Utilize financial strategies (9 strategies)**		
Access new funding	**1**	20%
Alter incentive/allowance structures	**0**	0%
Alter patient/consumer fees	**0**	0%
Develop disincentives	**0**	0%
Fund and contract for the clinical innovation	**0**	0%
Place innovation on fee for service lists/formularies	**0**	0%
Make billing easier	**0**	0%
Use capitated payments	**0**	0%
Use other payment schemes	**0**	0%
*Average of overall utilize financial strategies	**0.11**	2%
**Change infrastructure (8 strategies)**		
Change accreditation or membership requirements	**0**	0%
Change liability laws	**0**	0%
Change physical structure and equipment	**2**	40%
Change record systems	**3**	60%
Change service sites	**0**	0%
Create or change credentialing and/or licensure standards	**0**	0%
Mandate change	2	40%
Start a dissemination organization	**1**	20%
*Average of overall change infrastructure strategies	**1.0**	20%

*Average proportion utilizing strategies in each category, n = 5 sites

## Discussion

Assessment of implementation strategies is essential for identifying implementation barriers that should be addressed to improve the adoption, implementation and sustainability of an intervention [20].We highlight implementation strategies and outcomes used by hypertension programs occurring in five Lower-MIC participating in the NHLBI Hy-TREC network. The core implementation strategies being employed across the Hy-TREC programs are *use of evaluative and iterative strategies (i*.*e*., *needs assessment)*, *providing interactive assistance*, *adaptation and tailoring the intervention to context*, *develop stakeholder interrelationships*, *and providing training and education to stakeholders*. From the 73 ERIC’s suggested implementation strategies, the five hypertension research teams utilized 59 strategies, with use of financial incentives and change of infrastructure strategies being the least utilized. Because of the populations being engaged in these settings, it may have been difficult to include financial strategies in the intervention protocols as investigators may have been limited by funder /sponsor/ implementation setting restrictions. To enrich implementation effectiveness and improve clinical outcomes, it is recommended that adaptations are made to implementation strategies (i.e., training, financial and system change strategies) during implementation efforts in diverse settings [36, 40, 41]. Our findings conform to conclusions from existing literature assessing the use of implementation strategies in LMICs [42, 43]. The researchers note that multi-pronged implementation strategies are required to align interventions to health care systems care delivery models including a keen understanding of the local setting and context, whilst leveraging and adapting existing policies and payment systems [42, 43]. Also, providing professional training (knowledge translation), and empowerment of health care workers with necessary resources to carry out their duties [42, 43] is essential for improving implementation fidelity and successful intervention delivery. Essentially, effective implementation requires alignment of several factors, at the individual, community, provider, and health system level and interventions should be embedded within existing systems to have maximum reach [21]. Further, it is important to assess implementation outcomes including cost / cost effectiveness (financial impact of an implementation effort) to maximize resources especially in resource-constrained settings typical of Lower-MIC. Cost assessment should include costs of treatment delivery, cost of the implementation strategy, and cost of using the service in the particular setting [20]. Findings from existing systematic and narrative reviews emphasize the paucity of cost data measurement for interventions implemented in Lower-MIC [44, 45]. Of the five studies, three country sites are conducting a cost / cost effectiveness evaluation as part of their protocol.

Also, penetration (extent to which an innovation or practice is integrated within a service setting and its subsystems) [22] is necessary to ensure maximum reach of the intervention to needed populations, and is not directly being measured by sites as an outcome. Although we acknowledge that penetration and maintenance (sustainability) are related conceptually and empirically, and the majority of the program sites are measuring sustainability- which can provide insight into the degree of penetration. Also, all sites measured reach which informs the penetration and impact of an intervention or program [23]. Assessment of the penetration of an intervention should be included in future protocols to standardize the reporting of implementation outcomes. Without sufficient attention being paid to penetration, appropriateness (assessed by two countries), and implementation cost or financial impact of the implementation effort, we risk implementation success as these implementation outcomes capture contextual factors likely to influence the implementation process [22]. Also, an implementation success of an evidence-based intervention may be impacted by the extent of buy-in from organizational and political leadership. Health systems strengthening strategies for infrastructure change should be embedded in future interventions for hypertension control in Lower-MIC, although we recognize that there are practical drawbacks (i.e., difficulty incorporating infrastructure change strategies and feasibility issues) for Lower-MIC context. In certain context, existing political dynamics and hierarchical structure of the health care system create infrastructure change challenges. For example, in Ghana, community health nurses cannot prescribe antihypertensive medication; thus, an intervention that trains nurses to prescribe antihypertensives may be difficult to embed into the existing system. However, we have found that engaging key stakeholders (i.e., site leadership, health policymakers) from the onset and throughout the implementation process may encourage conversations around health policy changes and improve sustainable uptake of the intervention [46–48]. Nonetheless, the benefits / advantages of using context -sensitive implementation strategies include acceptability, adoption, and potential for long-term sustainability [6] and scale-up [49] of hypertension control interventions, especially those tailored to the implementation context. Some challenges of using the core implementation strategies include low physician-to-patient ratios and limited / lack of access to medications [1], complexity of the intervention, ability to embed the intervention into the existing clinical / organizational workflow, difficulty obtaining validated blood pressure devices, and improving provider / implementing staff/ patient knowledge about hypertension treatment and control [1, 50].

*Strengths and Limitations*: This study has a number of strengths including the use of data from multiple countries across three continents, making the findings generalizable across multiple geographical areas and across multiple health care settings such as those included in this study. Nonetheless it is critical to adapt and tailor any implementation strategy to fit the setting and population context to ensure implementation success [36]. Second, to the best of our knowledge, this is the first study to conduct a cross-country analysis of implementation strategies across multiple interventions for hypertension control in Lower-MIC using several implementation frameworks and assessing multiple implementation strategies and outcomes. As such, this comparison is unique and provides lessons learned in resource constrained environments with a significant burden of hypertension. We encourage other implementation scientist to conduct similar cross-country comparisons of interventions implemented in Lower-MIC and document lessons learned that can inform wider scale up of hypertension programs in that context. Third, the implementation strategy synthesis was guided by well-known ERIC strategies, with standard forms administered across sites. Our study will provide a significant amount of data for the community, and it will generate discussion on key implementation strategies that should be measured within Lower-MIC for hypertension control. We acknowledge the following limitations. First, early standardization of data collection for the main trials could improve future data harmonization across the various sites. Although we set out to conduct a data harmonization exercise, this was not feasible as site protocols were not standardized from the onset prior to initial implementation as a result of varying project start dates. Second, study design differences and varying context (e.g., health care systems) may impact the feasibility of using certain implementation strategies in a particular setting. For example, in some countries a task shifting / sharing approach is feasible; however, considering Ghana and India does not allow nurses to prescribe antihypertensive medication, a task shifting strategy where these professionals are trained to follow such protocol will be counterproductive and not well integrated into the existing system. Thus, context tailored interventions using context appropriate strategies will yield greater impact.

## Conclusion

Evidence from this study suggest that broad implementation strategies are incorporated into hypertension intervention protocols implemented in Lower-MIC. Although robust implementation strategies are being used in Lower-MIC, there is minimum use of financial and infrastructure change strategies. Our study contributes to the growing literature that demonstrates the use of implementation strategies to deliver evidence-based hypertension interventions in Lower-MIC and will inform future cross-country data harmonization activities in resource-constrained settings to improve comparability of study findings from diverse global settings.

## Supporting information

S1 Table Specifying implementation strategy (Example for main implementation components at each site). (PDF)

S1 File GRIT-CC data collection form. (PDF)
