# Supplementary material for: Implementation outcomes and strategies for delivering evidence-based hypertension interventions in lower-middle-income countries: Evidence from a multi-country consortium for hypertension control
Source: PLoS One. 2023 May 25;18(5):e0286204. doi: 10.1371/journal.pone.0286204 (PMC10212179; doi:10.1371/journal.pone.0286204)
Supplement: S1 Table — (PDF) [file pone.0286204.s001.pdf]

S1 Table 1. Specifying Implementation Strategy
(Example for main implementation components at each site)

Prerequisite	Ghana	Guatemala	India	Kenya	Vietnam
**Name it**	Practice facilitation and Task Shifting	Team based collaborative care	Task Shifting and Clinical decision support	Field Observations	Local needs assessment
**Define it**	Practice facilitation is an approach to supporting improvement in primary care practices that focuses on building organizational capacity for continuous improvement.<br>Task Shifting is a capacity building approach where non physician healthcare workers are trained to manage HTN patients	Healthcare personnel from health posts and health centers work collaboratively to establish a treatment plan for hypertensive patients	Task Shifting is a capacity building approach where non physician healthcare workers are trained to do non-essential/preliminary work of patient visit so that the physician can devote time in clinical decision making. Clinical decision support software- Guidelines based algorithms that generate clinical decision prompts for healthcare providers	Field observations of peer navigator actions	Collect and analyze data related to the need for the innovation
**Specify it (Operationalize it)**					
**The actor**	Community Health Nurses	Physicians, nurses and auxiliary nurses	Nonphysician and physician health care providers	Research staff	Stakeholders: local health leaders, health workers, community health workers (CHWs), patients with HTN
**The action**	Task-strengthening, i.e. Training community health workers in the 5A's (Ask, Assess, Advise, Assist, and Arrange)to identify counsel and refer adults with hypertension	Meetings to discuss cases of uncontrolled hypertension, and decide the course of action	Training non physician and physician health care providers on an integrated platform (eCRF and CDSS) to diagnose and manage hypertension and diabetes patients	Trained research staff will observe peer navigator actions and interactions during a site visit. The research staff is provided a checklist of expected behaviors and actions and will check off actions as they are completed	Carry out needs assessment (qualitative study) among stakeholders, Implement National HTN control program: Training health workers and CHWs to improve knowledge and skills in HTN management at community level

**Action Target**	Identify practice capacity for the adoption of TASSH in CHPS zones and develop a culturally tailored PF strategy using qualitative and quantitative methods. Also, we hope to observe a mean reduction in systolic blood pressure<br>- Unit of measurement<br>+ 12 months for intervention adoption<br>+ 24 months for intervention sustainability	Physicians, nurses and auxiliary nurses	Usage of the electronic case record form, appropriate NCD screening uptake, and adoption of the clinical decision support system.<br>Ultimately, we hope that this promotes guideline-based hypertension and diabetes management in this setting	Peer navigation (PNs)	Identify the gaps in HTN management and practice capacity for the adoption of study intervention and develop a culturally appropriate intervention using qualitative methods.<br>- Unit of measurement<br>+ 12 months for intervention adoption<br>+ 24 months for intervention sustainability
**Temporality**	Implementation Phase: When patients visit the CHPS compounds (after recruitment from home visits and health fairs)	Implementation Phase: Within the first month after enrollment begins	Implementation Phase	Implementation Phase: By month 3 of intervention	Implementation Phase
**Dose**	3 days every 6 months	At least once per month	Health care visits (frequency?)	1 day, continued if needed, based on peer performance	Needs assessment:<br>3 rounds (in years 1,3,5)
**Implementation outcome affected**	Adoption, Sustainability	Adoption	Adoption, Scalability	Fidelity	Adoption, Sustainability
