# Supplementary material for: Implementation outcomes and strategies for delivering evidence-based hypertension interventions in lower-middle-income countries: Evidence from a multi-country consortium for hypertension control
Source: PLoS One. 2023 May 25;18(5):e0286204. doi: 10.1371/journal.pone.0286204 (PMC10212179; doi:10.1371/journal.pone.0286204)
Supplement: S1 File — (PDF) [file pone.0286204.s002.pdf]

S1. File. GRIT-CC Data Collection Form

IMPLEMENTATION CONSTRUCTS

Table A. Implementation Framework by Study

	Guatemala	India	Ghana	Kenya	Vietnam
RE-AIM[1]					
Saunders[2]					
CFIR[3]					
PRECEDE-PROCEED[4,5]					
PRISM[6]					

Table B. Implementation Measures by Study

	Guatemala	India	Ghana	Kenya	Vietnam
Reach					
Effectiveness					
Adoption					
Sustainability					
Implementation					
Maintenance					
Acceptability					
Appropriateness					
Feasibility					
Fidelity					
Cost					
Penetration					
Dose					
Exposure					
Satisfaction					
Recruitment					
Context					
Implementation Climate					
Leadership Support					
Organizational Capacity					

Table C. Implementation Constructs as Defined by Site

Construct	General Definition	Definition by site	Information Source
Effectiveness			
Adoption			
Sustainability			
Acceptability			
Appropriateness			
Feasibility			
Fidelity			
Cost			
Penetration			
Recruitment			

IMPLEMENTATION STRATEGIES

Table D. Definition of Implementation Strategies

Activity	Definition	Used
Access new funding	Access new or existing money to facilitate the implementation	
Alter incentive/allowance structures	Work to incentivize the adoption and implementation of the clinical innovation	
Alter patient/consumer fees	Create fee structures where patients/consumers pay less for preferred treatments (the clinical innovation) and more for less-preferred treatments	
Assess for readiness and identify barriers and facilitators	Assess various aspects of an organization to determine its degree of readiness to implement, barriers that may impede implementation, and strengths that can be used in the implementation effort	
Audit and provide feedback	Collect and summarize clinical performance data over a specified time period and give it to clinicians and administrators to monitor, evaluate, and modify provider behavior	
Build a coalition	Recruit and cultivate relationships with partners in the implementation effort	
Capture and share local knowledge	Capture local knowledge from implementation sites on how implementers and clinicians made something work in their setting and then share it with other sites	
Centralize technical assistance	Develop and use a centralized system to deliver technical assistance focused on implementation issues	

Change accreditation or membership requirements	Strive to alter accreditation standards so that they require or encourage use of the clinical innovation. Work to alter membership organization requirements so that those who want to affiliate with the organization are encouraged or required to use the clinical innovation	
Change liability laws	Participate in liability reform efforts that make clinicians more willing to deliver the clinical innovation	
Change physical structure and equipment	Evaluate current configurations and adapt, as needed, the physical structure and/or equipment (e.g., changing the layout of a room, adding equipment) to best accommodate the targeted innovation	
Change record systems	Change records systems to allow better assessment of implementation or clinical outcomes	
Change service sites	Change the location of clinical service sites to increase access	
Conduct cyclical small tests of change	Implement changes in a cyclical fashion using small tests of change before taking changes system-wide. Tests of change benefit from systematic measurement, and results of the tests of change are studied for insights on how to do better. This process continues serially over time, and refinement is added with each cycle	
Conduct educational meetings	Hold meetings targeted toward different stakeholder groups (e.g., providers, administrators, other organizational stakeholders, and community, patient/consumer, and family stakeholders) to teach them about the clinical innovation	
Conduct educational outreach visits	Have a trained person meet with providers in their practice settings to educate providers about the clinical innovation with the intent of changing the provider's practice	
Conduct local consensus discussions	Include local providers and other stakeholders in discussions that address whether the chosen problem is important and whether the clinical innovation to address it is appropriate	
Conduct local needs assessment	Collect and analyze data related to the need for the innovation	
Conduct ongoing training	Plan for and conduct training in the clinical innovation in an ongoing way	
Create a learning collaborative	Facilitate the formation of groups of providers or provider organizations and foster a collaborative learning environment to improve implementation of the clinical innovation	
Create new clinical teams	Change who serves on the clinical team, adding different disciplines and different skills to make it more likely that the clinical innovation is delivered (or is more successfully delivered)	
Create or change credentialing and/or licensure standards	Create an organization that certifies clinicians in the innovation or encourage an existing organization to do so. Change governmental professional certification or licensure requirements to include delivering the	

	innovation. Work to alter continuing education requirements to shape professional practice toward the innovation	
Develop a formal implementation blueprint	Develop a formal implementation blueprint that includes all goals and strategies. The blueprint should include the following: 1) aim/purpose of the implementation; 2) scope of the change (e.g., what organizational units are affected); 3) timeframe and milestones; and 4) appropriate performance/progress measures. Use and update this plan to guide the implementation effort over time	
Develop academic partnerships	Partner with a university or academic unit for the purposes of shared training and bringing research skills to an implementation project	
Develop an implementation glossary	Develop and distribute a list of terms describing the innovation, implementation, and stakeholders in the organizational change	
Develop and implement tools for quality monitoring	Develop, test, and introduce into quality-monitoring systems the right input—the appropriate language, protocols, algorithms, standards, and measures (of processes, patient/consumer outcomes, and implementation outcomes) that are often specific to the innovation being implemented	
Develop and organize quality monitoring systems	Develop and organize systems and procedures that monitor clinical processes and/or outcomes for the purpose of quality assurance and improvement	
Develop disincentives	Provide financial disincentives for failure to implement or use the clinical innovations	
Develop educational materials	Develop and format manuals, toolkits, and other supporting materials in ways that make it easier for stakeholders to learn about the innovation and for clinicians to learn how to deliver the clinical innovation	
Develop resource sharing agreements	Develop partnerships with organizations that have resources needed to implement the innovation	
Distribute educational materials	Distribute educational materials (including guidelines, manuals, and toolkits) in person, by mail, and/or electronically	
Facilitate relay of clinical data to providers	Provide as close to real-time data as possible about key measures of process/outcomes using integrated modes/channels of communication in a way that promotes use of the targeted innovation	
Facilitation	A process of interactive problem solving and support that occurs in a context of a recognized need for improvement and a supportive interpersonal relationship	
Fund and contract for the clinical innovation	Governments and other payers of services issue requests for proposals to deliver the innovation, use contracting processes to motivate providers to deliver the clinical innovation, and develop new funding formulas that make it more likely that providers will deliver the innovation	

Identify and prepare champions	Identify and prepare individuals who dedicate themselves to supporting, marketing, and driving through an implementation, overcoming indifference or resistance that the intervention may provoke in an organization	
Identify early adopters	Identify early adopters at the local site to learn from their experiences with the practice innovation	
Increase demand	Attempt to influence the market for the clinical innovation to increase competition intensity and to increase the maturity of the market for the clinical innovation	
Inform local opinion leaders	Inform providers identified by colleagues as opinion leaders or "educationally influential" about the clinical innovation in the hopes that they will influence colleagues to adopt it	
Intervene with patients/consumers to enhance uptake and adherence	Develop strategies with patients to encourage and problem solve around adherence	
Involve executive boards	Involve existing governing structures (e.g., boards of directors, medical staff boards of governance) in the implementation effort, including the review of data on implementation processes	
Involve patients/consumers and family members	Engage or include patients/consumers and families in the implementation effort	
Make billing easier	Make it easier to bill for the clinical innovation	
Make training dynamic	Vary the information delivery methods to cater to different learning styles and work contexts, and shape the training in the innovation to be interactive	
Mandate change	Have leadership declare the priority of the innovation and their determination to have it implemented	
Model and simulate change	Model or simulate the change that will be implemented prior to implementation	
Obtain and use patients/consumers and family feedback	Develop strategies to increase patient/consumer and family feedback on the implementation effort	
Obtain formal commitments	Obtain written commitments from key partners that state what they will do to implement the innovation	
Organize clinician implementation team meetings	Develop and support teams of clinicians who are implementing the innovation and give them protected time to reflect on the implementation effort, share lessons learned, and support one another's learning	
Place innovation on fee for service lists/formularies	Work to place the clinical innovation on lists of actions for which providers can be reimbursed (e.g., a drug is placed on a formulary, a procedure is now reimbursable)	
Prepare patients/consumers to be active participants	Prepare patients/consumers to be active in their care, to ask questions, and specifically to inquire about care guidelines, the evidence behind clinical decisions, or about available evidence-supported treatments	
Promote adaptability	Identify the ways a clinical innovation can be tailored to meet local needs and clarify which elements of the innovation must be maintained to preserve fidelity	

Promote network weaving	Identify and build on existing high-quality working relationships and networks within and outside the organization, organizational units, teams, etc. to promote information sharing, collaborative problem-solving, and a shared vision/goal related to implementing the innovation	
Provide clinical supervision	Provide clinicians with ongoing supervision focusing on the innovation. Provide training for clinical supervisors who will supervise clinicians who provide the innovation	
Provide local technical assistance	Develop and use a system to deliver technical assistance focused on implementation issues using local personnel	
Provide ongoing consultation	Provide ongoing consultation with one or more experts in the strategies used to support implementing the innovation	
Purposely reexamine the implementation	Monitor progress and adjust clinical practices and implementation strategies to continuously improve the quality of care	
Recruit, designate, and train for leadership	Recruit, designate, and train leaders for the change effort	
Remind clinicians	Develop reminder systems designed to help clinicians to recall information and/or prompt them to use the clinical innovation	
Revise professional roles	Shift and revise roles among professionals who provide care, and redesign job characteristics	
Shadow other experts	Provide ways for key individuals to directly observe experienced people engage with or use the targeted practice change/innovation	
Stage implementation scale up	Phase implementation efforts by starting with small pilots or demonstration projects and gradually move to a system wide rollout	
Start a dissemination organization	Identify or start a separate organization that is responsible for disseminating the clinical innovation. It could be a for-profit or non-profit organization	
Tailor strategies	Tailor the implementation strategies to address barriers and leverage facilitators that were identified through earlier data collection	
Use advisory boards and workgroups	Create and engage a formal group of multiple kinds of stakeholders to provide input and advice on implementation efforts and to elicit recommendations for improvements	
Use an implementation advisor	Seek guidance from experts in implementation	
Use capitated payments	Pay providers or care systems a set amount per patient/consumer for delivering clinical care	
Use data experts	Involve, hire, and/or consult experts to inform management on the use of data generated by implementation efforts	
Use data warehousing techniques	Integrate clinical records across facilities and organizations to facilitate implementation across systems	
Use mass media	Use media to reach large numbers of people to spread the word about the clinical innovation	
Use other payment schemes	Introduce payment approaches (in a catch-all category)	

Use train-the-trainer strategies	Train designated clinicians or organizations to train others in the clinical innovation	
Visit other sites	Visit sites where a similar implementation effort has been considered successful	
Work with educational institutions	Encourage educational institutions to train clinicians in the innovation	

Note: Implementation Strategy definitions taken from Powell BJ, et al. (2015)[7].
Coding: "Y" – Implementation Strategy is used in the current study; "N" – Implementation strategy was not used in the current study; "?" – It is unclear whether the implementation strategy was used in the current study.

COMPONENTS OF IMPLEMENTATION STRATEGIES

Table E. Identifying components of the practice facilitation (PF) implementation strategy in Vietnam

Prerequisite	Requirements
Name it	
Define it	
**Specify it (Operationalize it)**	
The actor	
The action	
Action Target	
Temporality	
Dose	
Implementation outcome affected	
Justification	

REFERENCES

1. Glasgow RE, Vogt TM, Boles SM. Evaluating the public health impact of health promotion interventions: the RE-AIM framework. *Am J Public Health.* 1999;89(9):1322-1327.
2. Saunders RP, Evans MH, Joshi P. Developing a process-evaluation plan for assessing health promotion program implementation: a how-to guide. *Health Promot Pract.* 2005;6(2):134-147.
3. Damschroder LJ, Aron DC, Keith RE, Kirsh SR, Alexander JA, Lowery JC. Fostering implementation of health services research findings into practice: a consolidated framework for advancing implementation science. *Implement Sci.* 2009;4:50.
4. Crosby R, Noar SM. What is a planning model? An introduction to PRECEDE-PROCEED. *J Public Health Dent.* 2011;71:S7-S15.
5. Green L, Kreuter M. *Health program planning: An educational and ecological approach.* New York: McGraw-Hill; 2005.
6. Aqil A, Lippeveld T, Hozumi D. PRISM framework: a paradigm shift for designing, strengthening and evaluating routine health information systems. *Health Policy Plan.* 2009;24(3):217-228.
7. Powell BJ, Waltz TJ, Chinman MJ, et al. A refined compilation of implementation strategies: results from the Expert Recommendations for Implementing Change (ERIC) project. *Implement Sci.* 2015;10:21.
